# Attitudes toward artificial intelligence and the relationship between critical thinking tendencies and social intelligence: a study on sports science students

**DOI:** 10.3389/fpsyg.2026.1766712

**Published:** 2026-04-13

**Authors:** Ayşe Önal, Ahmet Dönmez, Engin Vural, Abdulkadir Yildiz, Yavuz Yildirim, Özgecan Ilgin

**Affiliations:** 1 Faculty of Sports Sciences, Afyon Kocatepe University, Afyonkarahisar, Türkiye; 2 Faculty of Sports Sciences, Alanya Alaaddin Keykubat University, Antalya, Türkiye; 3 Ministry of National Education, Bitlis, Türkiye; 4 Department of Recreation, Graduate School of Education, Sakarya University of Applied Sciences, Sakarya, Türkiye; 5 Independent Researcher, İzmir, Türkiye

**Keywords:** artificial intelligence, critical thinking, psychology, social intelligence, sports science student

## Abstract

This study aims to examine the associations between attitudes toward artificial intelligence, critical thinking tendencies, and social intelligence levels among students at the Faculty of Sports Sciences, and to investigate whether these variables differ according to gender and department of study. The participants in this quantitative research study, designed using a correlational survey model, consisted of 407 students (54.8% female, 45.2% male) enrolled in the 2025–2026 academic year in the departments of physical education and sports teaching, coaching education, and recreation at the Faculty of Sports Sciences of a state university. The data collection tools used were the “Personal Information Form,” the “Artificial Intelligence Attitude Scale,” the “Critical Thinking Tendency Scale (EMI),” and the “Tromsø Social Intelligence Scale.” In the statistical analysis of the data, independent samples *t*-test, one-way analysis of variance (ANOVA), Pearson correlation analysis, and linear regression analysis were used. When the findings were examined, no differences were found in students’ attitudes toward artificial intelligence, critical thinking tendencies, and social intelligence levels based on gender (*p* > 0.05); however, significant differences were found based on the variables of the department in which they were studying (*p* < 0.05). Furthermore, positive and significant relationships were found between attitudes toward artificial intelligence and critical thinking tendencies and social intelligence (*p* < 0.05). Moreover, it was revealed that attitudes toward artificial intelligence were significantly associated with critical thinking tendencies (*R*^2^ = 0.03, *p* < 0.05) and social intelligence (*R*^2^ = 0.04, *p* < 0.05), although the proportion of explained variance was relatively small. In conclusion, attitudes toward artificial intelligence are positively but modestly associated with critical thinking tendencies and social intelligence, and these findings should be interpreted cautiously given the cross-sectional design.

## 1 Introduction

One of the latest achievements in technology today is undoubtedly artificial intelligence. Artificial intelligence (AI) has entered our lives in many areas. Education, one of the first areas to be affected by all these developments, has also been influenced by AI. The rapid development of artificial intelligence and its effective role in many sectors has highlighted the need for education systems to integrate with this system and adapt quickly (Holmes et al., 2021).

It is clear that AI technology will lead to fundamental and modern changes in education. Therefore, there is a growing need for studies on the role of AI in education. Particularly in higher education, where the scope of AI application is expanding significantly, it is important to focus on the concepts associated with AI and what it positively or negatively affects. Considering that artificial intelligence is an important part of the future, it is evident that measuring students’ attitudes toward artificial intelligence is also important (Demir and Güraksın, 2022). In this context, this study seeks to answer questions such as: How does critical thinking, which is sought to be instilled in university students, relate to social intelligence, which plays an important role in establishing proper relationships with other individuals? How do certain demographic variables affect these relationships?

AI, which has now become one of our primary sources of reference, was first coined as a term by John McCarthy at a conference in 1956 (Jiang et al., 2022). AI is the ability of machines to cope with and adapt to emerging new situations, solve problems, answer questions, make certain device plans, and perform many other functions that humans would need to do using their intelligence (Bozkurt et al., 2021). In other words, it can be described as systems created by humans that mimic the human brain’s functions of thinking, reasoning, and learning, and can improve themselves by repeating the information they collect (Bozkurt et al., 2021; Obschonka and Audretsch, 2020). Although artificial intelligence has developed in many areas, its effects on education are still relatively new. It has begun to be used in education in recent years, but the adoption of artificial intelligence technology in education is closely related to students’ attitudes toward this technology. Therefore, determining students’ attitudes toward artificial intelligence is quite important (Alan et al., 2024).

The integration of AI into education is achieved by enabling the use of learning and teaching content that allows individuals to demonstrate their metacognitive skills (Shin, 2021). AI can make significant contributions to the field of education, particularly to Individualized Instruction Models (which include programmed learning/Skinner, individualized instruction/Keller plan, and computer-assisted instruction). Indeed, the common features of these models are independent learning, specific goals, active student participation, continuous assessment, remedial work, and the use of educational technologies (Köse, 2014). AI technology is well suited to achieving these pedagogical goals.

The innovations brought about by AI technology in the field of education are forcing people to keep pace with these developments. Accessing information is now very easy and fast. However, what is important is to analyze this information to reach correct and logical conclusions, develop different perspectives, and generate solutions to problems. This is only possible with advanced critical thinking skills. In this way, individuals can both realize their own potential and be respectful of others’ ideas. Indeed, Santos-Meneses et al. (2023) have recognized critical thinking skills as one of the most important skills of the 21st century. Critical thinking (CT) is defined as “logical and realistic thinking that enables a person to decide what to do and what to believe” (Ennis, 1987, p. 9). The skill of CR plays a critical role in helping individuals improve their academic achievement, solve problems in their lives, and adapt to the modern world and social life (Hwang et al., 2023; Vidal et al., 2023). From this perspective, many countries place great emphasis on developing critical thinking skills in their education systems (Alpizar et al., 2022). Possessing this skill is one of the factors that influence the careers of students, especially at the higher education level. At this stage, the following question comes to mind. How does the use of AI, which is so popular today, affect CT skills? The literature contains studies reporting positive results indicating that AI develops CT (Dwivedi et al., 2023; Nichols et al., 2024) and negative results indicating that it limits its development (Kasneci et al., 2023).

One of the skills university students need to achieve successful life and career goals is social intelligence. Social intelligence (SI), one of the areas in Gardner’s (2011) theory of multiple intelligences, is the ability to understand people’s emotions, attitudes, and behaviors, establish strong communication with them, recognize their talents, listen effectively, empathize, and build organizations. These skills encompass better understanding people and events, predicting possible developments, and steering events in the desired direction (Kurmanova et al., 2024). These characteristics are expected in a good teacher because teaching is the profession of educating people, and humans are social beings. Furthermore, possessing social intelligence can be considered the key not only to academic success but also to lifelong success. In particular, Students of the Faculty of Sports Sciences must be in constant communication with people as physical education teachers, coaches, and recreation leaders. This necessitates the development of their social intelligence. Social intelligence encompasses skills such as communicating with others and directing interactions. It is observed that the features offered by artificial intelligence to individuals, such as producing written and visual content, are increasingly integrated with social interaction and communication processes. Therefore, since individuals’ attitudes toward artificial intelligence are related to diversifying their interactions through technological tools and greater social integration, it is crucial to examine the relationship between social intelligence and attitudes toward artificial intelligence. When examining the relationship between AI and SI, some studies have highlighted the weakening of social skills among the risks associated with AI (Altun, 2024; Özalp, 2024).

As can be understood from the explanations, it is seen that the concepts of critical thinking and social intelligence may be related to artificial intelligence. As artificial intelligence technologies become central to everyday life, it is important for individuals to maintain and develop their cognitive and social capacities while adapting to these developments. The heavy use of technology-based applications in the education of sports science students highlights the importance of integrating artificial intelligence into their education. Furthermore, as these professions require high levels of communication and interaction, it is believed that this study will positively contribute to professional transformation in the field of sports science. In this regard, sports science students are a suitable population for examining the relationship between artificial intelligence attitudes, critical thinking, and social intelligence. Previous studies prior to the current study have generally examined attitudes toward artificial intelligence in terms of academic performance, technology acceptance, or digital literacy. However, studies that examine attitudes toward artificial intelligence within the same model alongside critical thinking and social intelligence structures are quite limited. The fact that these three variables have not been studied or have been studied only to a limited extent in relation to students of the Faculty of Sports Sciences represents a significant gap in the literature. Today, artificial intelligence technologies are increasingly transforming and developing learning processes and forms of social interaction. Revealing the relationship between attitudes toward artificial intelligence and social competencies and higher-order cognitive skills is important from an applied and institutional perspective. The present study aims to empirically examine this gap.

This study aims to examine the relationship between attitudes toward artificial intelligence and critical thinking tendencies and social intelligence levels among students at the Faculty of Sports Sciences, to determine the statistical predictive power of this attitude on the aforementioned variables, and to reveal whether the variables differ according to gender and department of study.

## 2 Methods

### 2.1 Research model

In this study, a correlational survey model, a type of survey model, was used to examine the relationships between attitudes toward artificial intelligence and critical thinking and social intelligence. The relational scanning model is defined as a scientific approach that aims to reveal the relationship between two or more quantitative variables (Fraenkel et al., 2012, p. 331). This model aims to examine correlational and statistical predictive relationships between variables, rather than causal ones. In this context, the study examined the extent to which attitudes toward artificial intelligence among sports science students are related to their critical thinking tendencies and social intelligence levels, and to what degree these variables are statistically predictive.

### 2.2 Research group

The population of the study consists of students enrolled in the undergraduate program of the Faculty of Sports Sciences at a state university. In the study, sampling was conducted by considering the sample size criteria accepted in the social sciences (*α* = 384) (Yazıcıoğlu and Erdoğan, 2014). Within this framework, a total of 407 sports science students, 223 of whom were female and 184 of whom were male, were included in the study using convenience sampling. Convenience sampling is defined in the literature as a method for “obtaining data quickly and cheaply” (Karagöz, 2017, p. 66). The use of this method has been considered a factor limiting the generalizability of the findings. Demographic information about the research participants is presented in Table 1.

**Table 1:** Demographic characteristics of the participants.

Variable	Category	*n*	%
Gender	Female	223	54.8
	Male	184	45.2
Department	Physical education and sports teaching	70	17.2
	Coaching education	167	41.0
	Recreation	170	41.8
Frequency of social activity participation	Never	10	2.5
	Rarely	99	24.3
	Occasionally	186	45.7
	Frequently	82	20.1
	Very frequently	30	7.4
	Total	407	100.0

The study included 407 university students. Detailed demographic distributions are presented in Table 1. The sample showed a relatively balanced distribution across departments, with a slight predominance of students from Recreation and Coaching Education programs. In general, participants reported occasional participation in social activities.

#### 2.2.1 Inclusion and exclusion criteria

##### 2.2.1.1 Inclusion criteria

The study included students:

- Who were enrolled in an undergraduate program at the Faculty of Sports Sciences during the data collection process,
- Who were 18 years of age or older.
- Who voluntarily agreed to participate in the study.

##### 2.2.1.2 Exclusion criteria

- Students not enrolled in the Faculty of Sports Sciences.
- Participants who completed the data collection tools incompletely or incorrectly were excluded from the study.

### 2.3 Data collection tools

#### 2.3.1 Personal information form

Within the scope of the study, a personal information form developed by the researchers was used to gather data from sports sciences students regarding gender, age, department of study, and frequency of participation in social activities.

#### 2.3.2 Artificial intelligence attitude scale

The Artificial Intelligence Attitude Scale developed by Aktay et al. (2024) was used in the study to measure participants’ attitudes toward artificial intelligence. The scale consists of 13 items designed to assess individuals’ perceptions of the benefits, risks, and usage tendencies related to artificial intelligence. Each item was rated on a five-point Likert scale ranging from “Strongly Disagree” to “Strongly Agree.” Items 4, 5, 6, and 7 in the scale were reverse-scored because they contained negative statements. This scale measures participants’ perceptions and attitudes toward artificial intelligence and does not directly assess the actual use, proficiency level, or frequency of use of artificial intelligence. In this context, the variable measured in the study is not artificial intelligence usage behavior or technological competence; it is the attitudinal tendency towards artificial intelligence. Therefore, the findings are based on participants’ cognitive and affective assessments of artificial intelligence, not on how often or to what extent they use it. The reliability analysis of the scale yielded 0.781 for the first factor (benefits of artificial intelligence), 0.732 for the second factor (risks of artificial intelligence), and 0.679 for the third factor (use of artificial intelligence). The reliability analysis for the scale as a whole was 0.802. In this study, the Cronbach’s alpha reliability coefficient of the scale was found to be 0.914 for the first factor, 0.855 for the second factor, 0.897 for the third factor, and 0.853 for the entire scale. In this study, evaluations of the scale were made based on total scores rather than sub-dimensions. When calculating the total score, the average of the item scores was taken to balance any differences that might arise from the number of items, and these average scores were used in the analyses.

#### 2.3.3 Critical thinking disposition scale

To determine the participants’ levels of critical thinking disposition, the “Critical Thinking Disposition Scale (EMI),” originally developed by Ricketts and Rudd (2005) and adapted into Turkish by Demircioğlu (2012), was used. The scale consists of 25 items structured on a five-point Likert format. Each item is scored as 5, 4, 3, 2, or 1, where a score of five indicates a high level of critical thinking disposition and a score of one indicates a low level. The reliability coefficients of the scale were reported as 0.84 for the “Engagement” subdimension, 0.71 for the “Cognitive Maturity” subdimension, 0.87 for the “Innovativeness” subdimension, and 0.88 for the total scale. In the current study, the reliability coefficients were found to be 0.932, 0.907, 0.907, and 0.967, respectively. In this research, evaluations related to the scale were conducted using the total score rather than subdimension scores. To eliminate potential differences resulting from the varying number of items, the mean item score was calculated, and these mean scores were used in the analyses.

#### 2.3.4 Tromso social intelligence scale

In this study, the “Tromsø Social Intelligence Scale,” originally developed by Silvera et al. (2001) and adapted into Turkish by Doğan and Çetin (2009), was used to assess participants’ levels of social intelligence. The scale consists of 20 items designed to evaluate individuals’ abilities to perceive, understand, and manage relationships in social contexts. Each item is scored on a five-point Likert scale ranging from “Not at all appropriate” (1) to “Completely appropriate” (5). Several items are reverse coded, specifically items 6, 9, 12, 14, 15, 16, 18, 19, and 20, which were scored inversely during evaluation. The reliability coefficient for the total score of the scale was reported as 0.83. For the subscales, the reliability coefficients were 0.77 for the “Social Information Processing” subscale, 0.84 for the “Social Skills” subscale, and 0.67 for the “Social Awareness” subscale. In the present study, the reliability coefficients were found to be 0.858, 0.718, and 0.788 for the respective subscales, and 0.818 for the total scale. In this research, evaluations related to the scale were conducted using the total score rather than subscale scores. To balance potential variations stemming from the different numbers of items, the mean item score was calculated and these mean scores were used in the analyses.

### 2.4 Data collection

Prior to the data collection process, the necessary approval was obtained from the Ethics Committee of a public university [Document Date and Number: 18.06.2025-371026]. Following ethical approval, Students of the Faculty of Sports Sciences were informed about the study and invited to participate voluntarily. The purpose of the research, principles of confidentiality, procedures for data use, and the voluntary nature of participation were explained to the students both in written and verbal form. It was emphasized that participation in the study was entirely voluntary. The data were collected through an online form that included the scales used in the study. At the beginning of the form, participants were asked to read and approve the informed consent statement, and access to the form was restricted for students who did not provide consent. The data collection process lasted approximately 2 weeks [13–24 October 2025], during which participant responses were obtained. After the data collection was completed, forms containing missing or incorrect information were excluded, and the remaining data were included in the analysis.

### 2.5 Data analysis

Normality analyses were performed to determine the distribution characteristics of the data in the study. For this purpose, the skewness and kurtosis values of the data were evaluated. In the analysis of skewness and kurtosis values, it was observed that skewness and kurtosis values for all variables ranged between ±2. Skewness and kurtosis values between −2 and +2 indicate that the data are normally distributed (George and Mallery, 2019, pp. 114–115). Parametric tests were preferred because the data showed a normal distribution. An independent samples t-test was applied for two-category variables, while a one-way analysis of variance (ANOVA) was applied for variables with three or more categories. Pearson correlation analysis was performed to examine the relationships between variables, and simple linear regression analysis was conducted to determine the statistical predictive relationships between variables.

## 3 Results

Since correlational and regression analyses were conducted using total scale scores rather than sub-dimension scores, internal consistency estimates for the total scores were calculated and reported. Cronbach’s alpha coefficients were 0.853 for the Artificial Intelligence Attitude Scale, 0.967 for the Critical Thinking Disposition Scale, and 0.818 for the Tromsø Social Intelligence Scale, indicating satisfactory to excellent internal consistency. Findings regarding whether students’ artificial intelligence attitudes, critical thinking dispositions, and social intelligence levels differ by gender are presented in Table 2.

**Table 2:** Comparison of artificial intelligence attitude, social ıntelligence, and critical thinking disposition by gender.

Variable	Gender	*n*	X̄	SS.	*t*	*p*
Artificial intelligence attitude	Female	223	3.23	0.61	−0.434	0.665
	Male	184	3.25	0.63		
Critical thinking disposition	Female	223	3.45	0.68	−1.546	0.123
	Male	184	3.56	0.72		
Social intelligence	Female	223	3.33	0.39	−0.160	0.873
	Male	184	3.33	0.50		

As presented in Table 2, no statistically significant gender differences were observed in artificial intelligence attitude, critical thinking disposition, or social intelligence scores. Overall, female and male students demonstrated comparable levels across all examined variables.

Findings regarding whether students’ artificial intelligence attitudes, critical thinking dispositions, and social intelligence levels differ by academic department are presented in Table 3.

**Table 3:** Anova results for artificial ıntelligence attitude, social ıntelligence, and critical thinking disposition by academic department.

Variable	Department	*n*	X̄	SS	*F*	*p*	Difference
Artificial intelligence attitude	Physical education and sports teachingᵃ	70	3.42	0.57	4.154	0.016	a-b
	Coaching educationᵇ	167	3.16	0.67			
	Recreationᶜ	170	3.24	0.58			
Critical thinking disposition	Physical education and sports teachingᵃ	70	3.69	0.77	3.101	0.046	a-b,c
	Coaching educationᵇ	167	3.46	0.68			
	Recreationᶜ	170	3.45	0.68			
Social intelligence	Physical education and sports teachingᵃ	70	3.35	0.42	0.127	0.881	
	Coaching educationᵇ	167	3.33	0.43			
	Recreationᶜ	170	3.32	0.46			

According to the results presented in Table 3, the mean artificial intelligence attitude score of students in the Physical Education and Sports Teaching program was 3.42, compared to 3.16 in the Coaching Education program and 3.24 in the Recreation program. A one-way ANOVA indicated that the difference between the groups was statistically significant (*F*_(2, 404)_ = 4.154, *p* = 0.01). *Post hoc* analysis using the Scheffé test showed that students in the teaching program had significantly higher scores than those in the coaching program. With regard to critical thinking disposition, a significant difference was also found among the departments (*F*_(2, 404)_ = 3.101, *p* = 0.046). The Scheffé *post hoc* test revealed that students in the teaching program scored significantly higher than students in both the coaching and recreation programs. In terms of social intelligence, no significant difference was detected between departments (*F*_(2, 404)_ = 0.127, *p* = 0.881). This indicates that students’ social intelligence levels do not differ statistically across academic departments.

Findings regarding the relationships between students’ artificial intelligence attitudes and their critical thinking dispositions and social intelligence levels are presented in Table 4.

**Table 4:** Findings from the Pearson correlation analysis.

Variable	Statistic (r, p)	Social intelligence	Critical thinking
Artificial intelligence attitude	*r*	0.196^**^	0.167^**^
	*p*	0.000	0.001

According to the Pearson correlation analysis presented in Table 4, a positive and low-level significant relationship was found between artificial intelligence attitude and social intelligence (*r* = 0.196, *p* = 0.000). Additionally, a positive and low-level significant relationship was identified between artificial intelligence attitude and critical thinking disposition (*r* = 0.167, *p* = 0.001).

Finally, simple linear regression analysis was conducted to determine the extent to which Sports Sciences Faculty students’ attitudes toward artificial intelligence predict their critical thinking dispositions and social intelligence levels. The findings obtained from these analyses are presented in Table 5.

**Table 5:** Regression analysis results for the association of artificial intelligence attitude with critical thinking and social intelligence.

Predictor	Critical thinking				Social intelligence			
	B	SE	*β*	*p*	B	SE	*β*	*p*
Constant	2.887	0.183	—	<0.001	2.882	0.115	—	<0.001
Artificial intelligence attitude	0.189	0.055	0.167	0.001	0.140	0.035	0.196	<0.001

*N* = 407. Enter method was used. For Critical Thinking: *R* = 0.17, *R*^2^ = 0.03, *F*_(1,405)_ = 11.662, *p* = 0.001.

For social intelligence: *R* = 0.20, *R*^2^ = 0.04, *F*_(1,405)_ = 16.236, *p* < 0.001.

The results of the linear regression analysis indicated that attitudes toward artificial intelligence were positively and significantly associated with critical thinking disposition (*β* = 0.167, *t* = 3.415, *p* = 0.001). The model explained approximately 3% of the variance in critical thinking (*R*^2^ = 0.03). The calculated effect size was *f*^2^ = 0.031, which corresponds to a small effect according to Cohen’s (1988) criteria. Although the relationship was statistically significant, its practical magnitude appears limited. Similarly, attitudes toward artificial intelligence were positively and significantly associated with social intelligence (*β* = 0.196, *t* = 4.029, *p* < 0.001). The model accounted for approximately 4% of the variance in social intelligence (*R*^2^ = 0.04). The corresponding effect size was *f*^2^ = 0.042, which is also classified as small based on Cohen’s (1988) benchmarks. These findings suggest that while the associations are statistically significant, the amount of explained variance indicates modest practical impact.

## 4 Discussion and conclusion

This study aims to examine the relationship between attitudes toward artificial intelligence and critical thinking tendencies and social intelligence levels among students at the Faculty of Sports Sciences, to determine the statistical predictive power of this attitude on the aforementioned variables, and to reveal whether the variables differ according to gender and department of study.

The first finding regarding demographic variables in the study relates to gender. No significant differences were found in any of the three concepts in the gender variable. Other studies have found that in the technical understanding dimension of artificial intelligence literacy, male students (Elçiçek, 2024) tend to think more critically than female students (Gülle, 2015; Mutlu et al., 2016), while male students score significantly higher in social intelligence (Durukan et al., 2020). These studies in the literature differ from the present study. This may be due to the fact that the concepts in question vary in terms of gender across different fields.

The findings obtained in the variable of the department in which Students of the Faculty of Sports Sciences study show that students in the physical education and sports teaching department differ significantly from students in the coaching and recreation departments in terms of their attitudes toward artificial intelligence and critical thinking tendencies. This finding highlights that education department students have higher artificial intelligence attitudes and critical thinking tendencies than students in other departments. The increased discussion of pedagogical reflection and technology adaptation in teacher education programs may be a cognitive and pedagogical explanation for the existing difference. In our country, the department with the highest admission score for sports science faculties is the teacher training department. Therefore, students studying in this department are likely to have higher academic achievement compared to other departments. Studies in the literature indicating that critical thinking skills develop as academic achievement increases (Alsancak and Aybek, 2023; Canto et al., 2021; Mutlu and Çetinkaya, 2023; Orhan, 2022) support this finding. There are differing views on the impact of artificial intelligence (AI) on academic achievement. For example, Wei et al. (2021) and Tiwari (2023) state in their studies that the use of AI in higher education will lead to high academic achievement due to the personalized learning support it provides. However, Bender (2024) and Xiaolei and Teng (2024) have expressed the view that the use of AI hinders the development of cognitive skills due to the risk of providing students with incorrect feedback and presenting information ready-made. Since academic achievement was not measured in this study, the studies in the literature on the relationship between artificial intelligence and critical thinking and academic achievement are considered only as an explanatory framework. No significant differences were found in the Social Intelligence scores of the departments within the Faculty of Sports Sciences. This finding can be considered an indication that students studying in the field of sports sciences possess similar levels of social intelligence.

The study reveals that students in the Faculty of Sports Sciences exhibit a positive and low-level significant relationship between their attitudes toward artificial intelligence and their critical thinking tendencies and social intelligence. These findings indicate that there is a positive relationship between Students of the Faculty of Sports Sciences attitudes toward artificial intelligence and their critical thinking tendencies and social intelligence levels. However, this relationship reflects statistical association between the variables rather than a causal increase.

Studies examining the positive and negative relationship between the use of Artificial Intelligence tools in education and the concepts of Critical Thinking and Social Intelligence have been reviewed in the literature. First, studies reporting positive results between Artificial Intelligence and Critical Thinking Tendencies were examined (Altun, 2024; Chaparro-Banegas et al., 2024; Çavuş, 2024; Dwivedi et al., 2023; Essien et al., 2024; Nichols et al., 2024; Özalp, 2024; Saulius and Malinauskas, 2021), revealing that AI tools play a complementary and developmental role in critical thinking skills. In these studies, artificial intelligence supports personalized learning, enabling students to participate in the learning process at their own pace and with active engagement; AI tools offering complex problems and interactive games that give students the opportunity to try different approaches when solving problems; It is argued that the broad-based information provided by AI can develop critical thinking skills by presenting different theories and perspectives. Studies reporting negative results between Artificial Intelligence and Critical Thinking Tendencies have stated that Artificial Intelligence may limit and weaken critical thinking skills (Dwivedi et al., 2023; Kasneci et al., 2023; Özalp, 2024). These studies also highlight the risk that the ready-made information provided by Artificial Intelligence may make students lazy. It is stated that students may be able to obtain information without questioning it, drift away from conducting their own research and reaching original conclusions, and that these factors may hinder their critical thinking skills. The positive relationship found in this study can be explained by students using artificial intelligence not primarily to access information, but rather for curiosity and exploration. The variable measured in this study is not artificial intelligence usage behavior or artificial intelligence competence, but rather attitudes toward artificial intelligence. The concept of attitude reflects an individual’s cognitive and affective evaluations of a particular phenomenon, while actual use and competence are based on behavioral indicators. Therefore, the findings reveal the statistical relationship between perceptions and evaluation tendencies toward artificial intelligence and critical thinking and social intelligence, rather than the level of artificial intelligence use.

In the literature, negative results have been observed between Artificial Intelligence and Social Intelligence. Koçyiğit and Darı (2023) stated that Artificial Intelligence cannot behave like a social entity in the communication process because it lacks empathy and emotion. Sap et al. (2022) also examined the state of social intelligence and Theory of Mind in Natural Language Processing (NLP) systems such as Chat CPT in their studies and found that the results were significantly below human performance. There are also studies indicating that the excessive use of Artificial Intelligence can weaken social skills (McGuire et al., 2024; Özalp, 2024). These results do not correspond with the positive results found in the study between Artificial Intelligence and Social Intelligence. This may be attributed to differences in the sample. Sports science students have a higher level of face-to-face social interaction than students studying other subjects due to their participation in sports. The fact that their social skills are functionally relevant in a professional context reinforces the view that their attitudes toward artificial intelligence positively influence their social intelligence. However, this situation is merely a possible explanation based on the structural characteristics of the sample rather than causality.

Low *R*^2^ values indicate that attitudes toward artificial intelligence have a limited effect on the critical thinking and social intelligence analyzed. Small effect sizes are commonly observed in the social sciences, particularly in the relationships between psychosocial variables (Funder and Ozer, 2019). Low *R*^2^ values may be related to the multidimensional and factorial structure of critical thinking and social intelligence. Many similar variables, such as an individual’s pedagogical experience, social environment, cognitive capacity, and personality traits, can influence these skills. From this perspective, it should be noted that attitudes toward artificial intelligence are not the only factor influencing these skills but rather one of the contributing factors. According to Rosenthal and Rubin (1982), when the practical consequences indicated by some seemingly minor statistical relationships are evaluated, they can provide significant insights. From this perspective, especially when large samples are considered, low correlation coefficients can reveal meaningful and concrete results. Rosenthal (1990) calculated in his study that a correlation coefficient of approximately 0.03 regarding aspirin use after a heart attack in a sample of 10,845 people could prevent a possible heart attack in 85 people. Looking at this example, it is important to note that although the effect is statistically small at the societal level, it could yield noteworthy results. In a study involving over 2,000 participants and 2 million financial transactions, a correlation coefficient of 0.09 was found between a participant’s extraversion score and the amount spent on vacation (Weston et al., 2018). It is clear that a correlation value that is insignificant for an individual can contribute significantly to the literature for a large sample study. Abelson (1985) emphasized that effects observed at low levels may not be decisive at first, but can produce meaningful results when spread across a large population or repeated over time. Effects at low levels can yield noteworthy results, particularly in highly variable and complex fields such as psychology, through social outcomes and long-term behaviors.

The current study has some limitations. First, the research consists of students who are continuing their education at the Faculty of Sports Sciences of a single state university and who were selected using convenience sampling. This situation may limit the generalization of the findings to different universities, interdisciplinary and cultural contexts. Therefore, the results presented should be interpreted for student groups with similar demographic and academic characteristics. It is recommended that future studies use samples selected from different universities, different departments, and probability sampling methods to strengthen external validity.

### 4.1 Recommendations

This study found that the relationship between sports science students’ attitudes toward artificial intelligence and their critical thinking tendencies and social intelligence is positive but low. In this context, providing interdisciplinary supportive education, developing artificial intelligence-based feedback systems, and incorporating them into tactical analyses and performance evaluation processes may influence attitudes toward artificial intelligence. It is thought that providing education on the limitations, risks, and purposes of artificial intelligence may be important in terms of increasing awareness of artificial intelligence.

Future studies conducted with different samples may yield different results in identifying the links between attitudes toward artificial intelligence, critical thinking tendencies, and social intelligence disciplines. Longitudinal studies can examine changes in attitudes over time. It is thought that future studies could provide a stronger explanation of cognitive processes in models guided by artificial intelligence adequacy and artificial intelligence literacy in real-world usage behaviors.

## Data Availability

The raw data supporting the conclusions of this article will be made available by the authors, without undue reservation.
